# The global patient-reported outcomes for multiple sclerosis initiative: bridging the gap between clinical research and care – updates at the 2023 plenary event

**DOI:** 10.3389/fneur.2024.1407257

**Published:** 2024-06-20

**Authors:** Paola Zaratin, Sara Samadzadeh, Meral Seferoğlu, Vito Ricigliano, Jonadab dos Santos Silva, Abdulkadir Tunc, Giampaolo Brichetto, Timothy Coetzee, Anne Helme, Usman Khan, Robert McBurney, Guy Peryer, Helga Weiland, Peer Baneke, Mario Alberto Battaglia, Valerie Block, Luca Capezzuto, Loïc Carment, Paolo Angelo Cortesi, Gary Cutter, Letizia Leocani, Hans-Peter Hartung, Jan Hillert, Jeremy Hobart, Kaisa Immonen, Paul Kamudoni, Rod Middleton, Patricia Moghames, Xavier Montalban, Liesbet Peeters, Maria Pia Sormani, Susanna van Tonder, Angela White, Giancarlo Comi, Patrick Vermersch

**Affiliations:** ^1^Research Department, Italian Multiple Sclerosis Foundation, Genoa, Italy; ^2^Charité – Universitätsmedizin Berlin, Corporate Member of Freie Universität Berlin and Humboldt-Universität zu Berlin, Experimental and Clinical Research Center, Berlin, Germany; ^3^Institute of Regional Health Research and Molecular Medicine, University of Southern Denmark, Odense, Denmark; ^4^Department of Neurology, The Center for Neurological Research, Næstved-Slagelse-Ringsted Hospitals, Slagelse, Denmark; ^5^Department of Neurology, Bursa Faculty of Medicine, Bursa Yüksek İhtisas Training and Research Hospital, University of Health Sciences, Bursa, Türkiye; ^6^Sorbonne Université, Paris Brain Institute, ICM, CNRS, Inserm, Paris, France; ^7^Neurology Department, Pitié-Salpêtrière Hospital, APHP, Paris, France; ^8^Department of Neurology, Icahn School of Medicine at Mount Sinai, New York, NY, United States; ^9^Programa de Pós Graduação Stricto Senso em Neurologia, Department of Neurology, Fluminense Federal University, Niterói, Brazil; ^10^Department of Neurology, Sakarya University Faculty of Medicine, Sakarya, Türkiye; ^11^National Multiple Sclerosis Society, New York, NY, United States; ^12^Multiple Sclerosis International Federation, London, United Kingdom; ^13^Institute for Healthcare Policy, KU Leuven, Leuven, Belgium; ^14^Accelerated Cure Project, Waltham, MA, United States; ^15^Multiple Sclerosis Society UK, London, United Kingdom; ^16^Multiple Sclerosis South Africa, Hermanus, Western Cape, South Africa; ^17^Department of Life Science, University of Siena, Siena, Italy; ^18^University of California, San Francisco, San Francisco, CA, United States; ^19^Hoffmann-La Roche, Basel, Switzerland; ^20^Ad Scientiam, Paris, Île-de-France, France; ^21^Research Centre on Public Health (CESP), University of Milan-Bicocca, Milan, Italy; ^22^Department of Biostatistics, School of Public Health, The University of Alabama at Birmingham, Birmingham, AL, United States; ^23^University Vita-Salute San Raffaele, Milan, Italy; ^24^Department of Rehabilitation Sciences, Casa di Cura Igea, Milan, Italy; ^25^Department of Neurology, UKD, Medical Faculty, Heinrich Heine Universitat Düsseldorf, Düsseldorf, Germany; ^26^Brain and Mind Center, University of Sydney, Camperdown, NSW, Australia; ^27^Department of Neurology, Medical University of Vienna, Vienna, Austria; ^28^Department of Neurology, Palacky University Olomouc, Olomouc, Czechia; ^29^Department of Clinical Neuroscience, Neurogenetics Multiple Sclerosis, Karolinska Institutet, Stockholm, Sweden; ^30^Plymouth University Peninsula Schools of Medicine and Dentistry Devon, Plymouth, United Kingdom; ^31^European Medicines Agency, Public and Stakeholder Engagement Department, Amsterdam, North Holland, Netherlands; ^32^Merck KGaA, Darmstadt, Germany; ^33^Faculty of Medicine Health and Life-Sciences, Swansea University, Swansea, United Kingdom; ^34^European MS Platform, Brussels, Belgium; ^35^Hopital Vall d’Hebron, Universitat Autonoma de Barcelona, Barcelona, Spain; ^36^Hasselt University–Biomedical Research Institute (BIOMED), Hasselt, Belgium; ^37^Department of Health Sciences, University of Genoa, Genoa, Italy; ^38^MS Lëtzebuerg, Luxembourg, Belgium; ^39^Université de Lille, Inserm LilNCog, CHU Lille, FHU Precise, Lille, France

**Keywords:** multiple sclerosis progression, patient reported outcomes, patient engagement, personalized medicine, digital health

## Abstract

Significant advancements have been achieved in delineating the progress of the Global PROMS (PROMS) Initiative. The PROMS Initiative, a collaborative endeavor by the European Charcot Foundation and the Multiple Sclerosis International Federation, strives to amplify the influence of patient input on MS care and establish a cohesive perspective on Patient-Reported Outcomes (PROs) for diverse stakeholders. This initiative has established an expansive, participatory governance framework launching four dedicated working groups that have made substantive contributions to research, clinical management, eHealth, and healthcare system reform. The initiative prioritizes the global integration of patient (For the purposes of the Global PROMS Initiative, the term “patient” refers to the people with the disease (aka People with Multiple Sclerosis – pwMS): any individual with lived experience of the disease. People affected by the disease/Multiple Sclerosis: any individual or group that is affected by the disease: E.g., family members, caregivers will be also engaged as the other stakeholders in the initiative). insights into the management of MS care. It merges subjective PROs with objective clinical metrics, thereby addressing the complex variability of disease presentation and progression. Following the completion of its second phase, the initiative aims to help increasing the uptake of eHealth tools and passive PROs within research and clinical settings, affirming its unwavering dedication to the progressive refinement of MS care. Looking forward, the initiative is poised to continue enhancing global surveys, rethinking to the relevant statistical approaches in clinical trials, and cultivating a unified stance among ‘industry’, regulatory bodies and health policy making regarding the application of PROs in MS healthcare strategies.

## 1 Introduction

### 1.1 The global patient-reported outcomes for multiple sclerosis initiative

**Figure 1:**
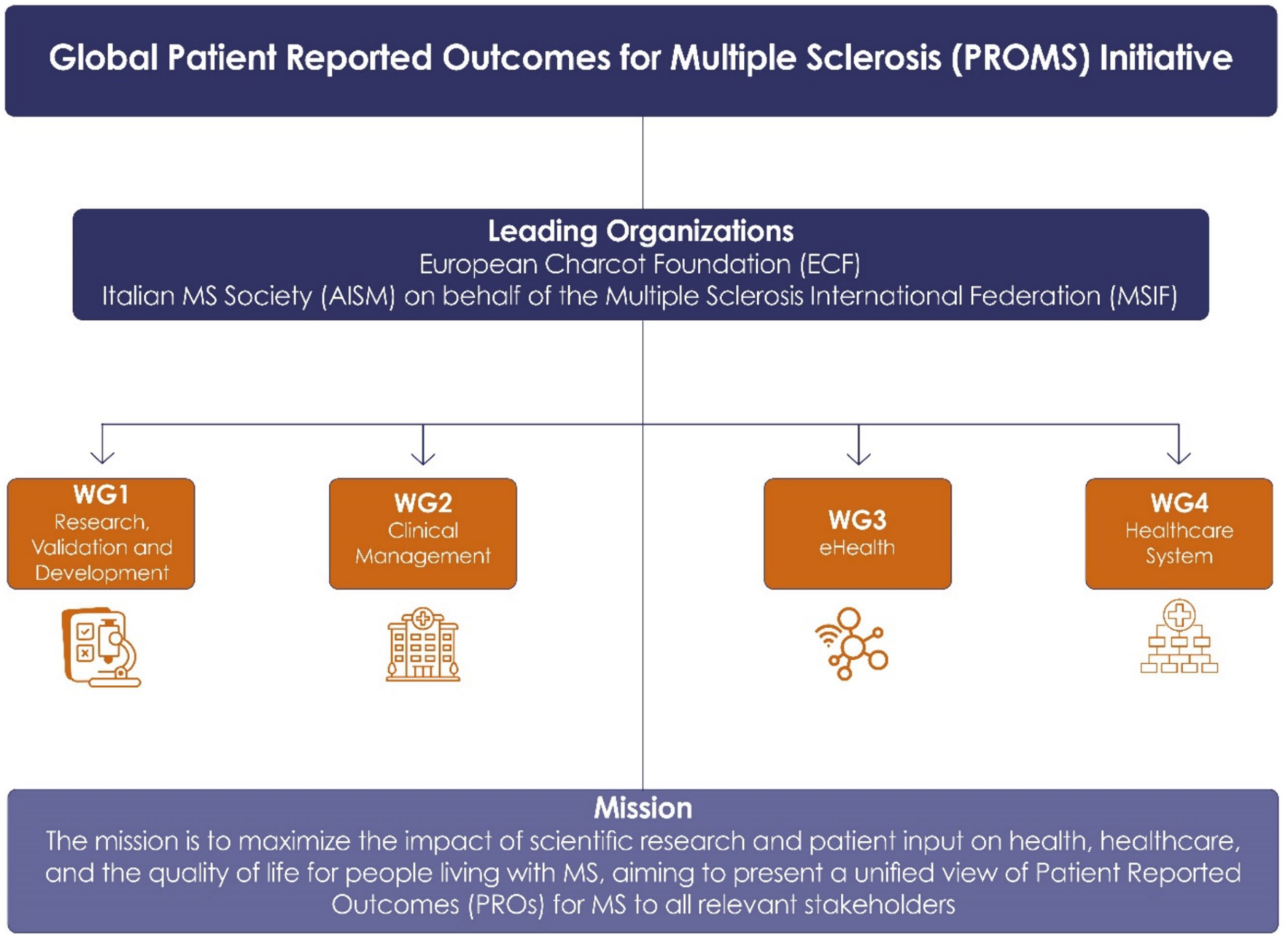
Structure of the Global PROMS Initiative, illustrating the collaboration among leading MS organizations and the division into four working groups focused on research, clinical management (care), eHealth, and healthcare systems to fulfill the mission of the PROMS Initiative.

The global Patient Reported Outcome for Multiple Sclerosis (PROMS) Initiative^1^ was inaugurated on 12 September 2019 at the 35th Congress of the European Committee for Treatment and Research in Multiple Sclerosis (ECTRIMS). This multi-stakeholder PROMS Initiative is jointly led by the European Charcot Foundation (ECF) and the Multiple Sclerosis International Federation (MSIF) with the Italian MS Society acting as lead agency for and behalf of the Global MSIF Movement (1). It includes 60 international experts from various stakeholder categories, including (patients, patient organizations, industry, research/clinician, healthcare organizations and health economists). The initiative was founded upon an open and participatory governance structure (Figure 1). Four distinct working groups (WG) have been formed: (1) Research, Validation, and Development, focusing on advancing clinical research innovation; (2) Clinical Management, dedicated to improving the existing patient outcomes; (3) eHealth, exploring digital technology integration in healthcare; and (4) Healthcare System, which is centered on shaping and adapting healthcare systems to meet future challenges effectively.

The mission of this initiative is: (1) to maximize scientific research and patient input on health, healthcare and the quality of life of people living with MS (see Figure 1), and (2) to represent a unified view on Patient-Reported Outcomes for MS (PROs) to people with MS (pwMS), healthcare providers, regulatory agencies and Health Technology Assessment agencies (HTAs).

To fulfill its mission PROMS’s strategic direction is engaging pwMS in providing Patient-Reported Outcomes Measures (PROMs) that give us a picture of their status today and changes over time, leveraging passive and active monitoring, in a holistic, scientifically sound, comprehensive and personalized approach to improve prognosis, prevent progression and improve lives of people living with MS.

This strategy is aligned with the European Commission’s^2^ increasing demand for an increased engagement of pwMS in research to promote the paradigm shift toward predictive, preventive, and personalized medicine (2). By emphasizing the need for a renewed concept of personal value-based care approach, the initiative ensures that patient values are at the core of decision-making processes from research to care. Furthermore, digital technology plays a vital role in this paradigm shift, facilitating the incorporation and acknowledgment of perspectives from those living with the disease over time, thus bolstering this transformative approach in healthcare.

### 1.2 Engagement coordination team

An Engagement Coordination Team (ECT) forms a crucial component within the governance structure of the initiative. The ECT is dedicated to maximizing outreach efforts, aiming to engage with the broadest possible spectrum of the global MS community and to bring pwMS experiential knowledge in the WGs’ agenda. The concept of ECT originated from the EU-funded MULTI-ACT project (3), which was designed to ensure representation and inclusiveness of the relevant MS community. In alignment with the initiative, integrating PROMs into patient care offers a unified approach that surpasses geographical limitations. This unified approach enhances international collaboration, research, and best practice exchange, ultimately improving patient care worldwide.

### 1.3 Progress and prospects: navigating the PROMS initiative phases

Phase 1 of the initiative involves understanding the state-of-the-art global landscape of PROMs for MS through four working groups (WG1 to WG4). WG1 explores which outcomes matter most to pwMS, while WG2 examines the current use of PROMs in clinical care. WG3 investigates existing eHealth tools and technology, and WG4 assesses the influence of PROMs on policy for MS healthcare. The phase concludes with an impact assessment of participatory governance, which includes widening collaborations, establishing partnerships, and raising awareness.

In Phase 2 of the PROMS Initiative, which took place at a plenary event on November 8th, 2023, in Baveno, Italy, the focus was on optimizing the impact of PROMs in the clinical research and care of MS patients. The session explored the added value of PROMs in identifying unrecognized progression and discussed whether this could establish PROMs as a primary endpoint for treatments. It also addressed the challenges of transitioning PROMs from clinical trials to practical care settings, and how the initiative’s achievements are addressing these challenges, contributing to the success of the efforts. The commitment to continue working on these fronts was reaffirmed, indicating an ongoing effort to refine and enhance the use of PROMs in MS management.

## 2 Role of PROMs and patient generated data in highlighting “unrecognized” progression

Patient-Reported Outcomes (PROs) are defined as any direct report from patients about the status of their health condition, without the interpretation of their responses by clinicians or anyone else. This concept was outlined by the Food Drug Administration (FDA) in 2009^3^ and similarly, by the European Medicines Agency (EMA) in 2014.^4^ However, PROMs refer to any tools, like questionnaires or instruments, that record health-related data collected from patients’ self-reporting, which can include both active and passive input. These definitions underscore the importance of the patient’s voice in assessing health outcomes and the effectiveness of medical interventions.

The concept of progression in MS varies depending on the perspective. Clinical trials may define progression through objective measures such as confirmed disability worsening assessed by the expanded disability status scale (EDSS) and quantitative MRI techniques in successive visits, a significant increase in timed walk as measured by Timed 25-Foot Walk and 9 Hole Peg Test time, or a decrease in cognition as measured by Symbol Digit Modalities Test (SDMT) scores. Clinicians might assess progression through the stage of a disease, noting sustained worsening of functions or symptoms that were previously under control. Patients, on the other hand, may perceive progression as an increased cumulative impact of disease progression, concerns regarding long term medication or concerns relating to later life. This demonstrates the different nature of disease progression and the importance of considering multiple viewpoints in healthcare.

The discourse on MS progression has evolved to acknowledge forms of deterioration that are subtle or challenging to detect. Key terms such as “silent progression,” “PIRA” (Progression Independent of Relapse Activity), and “unrecognized progression” have emerged in recent literature to describe this phenomenon (4, 5) These concepts highlight a critical aspect of MS: significant but insidious worsening that may manifest early and evade detection by conventional metrics (5–9).

The current clinical trial tools for MS, including the EDSS and MS Functional Composite (MSFC), persist from past decades despite recognized limitations. The EDSS measure is biased toward ambulation, misses subtle differences within disabilities, and fails to capture the full spectrum of patient activity and community participation. Other measures such as MSFC, No Evidence of Disease Activity (NEDA), and various quantitative MRI techniques, though advantageous, have their constraints due to availability and challenges in objective measurement (10, 11). The MSFC, for instance, omits bladder function, a critical quality-of-life aspect, due to the absence of a reliable measure. Similarly, minor EDSS changes from a 1.5 to a 2 can have profound implications for a patient’s quality of life (QoL), often overlooked in clinical trials (12).

Overall, the existing clinical outcomes provide a periodic functional snapshot in a limited number of domains or symptoms of importance to people affected by MS. Identifying both subjective and objective measures of functional domains and monitoring MS fluctuations over time will help interventions and treatments for MS overcome the hurdles of phase II and phase III, securing broader access to care.

There is increasing advocacy for PROMs, Patient-Reported Experience Measures (PREMs), and Patient Preference Information (PPI) which encompass active and passive data collection on functional domains of MS. These patient-generated data are gaining traction for their potential to enrich traditional measures and quantify the patient experience more precisely. Despite this, integrating PROMs into clinical trials and care faces challenges, necessitating consensus among stakeholders, including regulatory bodies, the pharmaceutical industry, clinicians, and insurers.

The adoption of more sophisticated metrics in clinical research is unfolding progressively. Regulatory bodies, such as the FDA, increasingly acknowledge the significance of PROMs for gauging treatment efficacy. This shift is exemplified by the acceptance of passive PROMs, like the use of Fitbit® devices, as exploratory outcome measures in studies such as SPI2 (13). This change also reflects a growing appreciation for the lived experiences of people with chronic conditions.

The SPI2 study, a phase 3 international placebo-controlled randomized control trial, was likely the first of its kind to incorporate remote activity monitoring as an exploratory outcome measure. This study aimed at evaluating the efficacy and safety of high-dose biotin in people with non-active progressive MS. Despite being a negative trial, with no significant difference between placebo and treatment groups, it revealed a decrease in steps in participants with worsening EDSS scores and slower 25-foot walk times. The successful implementation of remote activity monitoring in this large, international, multicenter, multi-language randomized trial with over 600 progressive pwMS was a significant milestone, showcasing the feasibility and value of such methods in extensive clinical research settings (13).

Similar to SPI2 trial, in another example of passive continuous monitoring of ambulatory function in MS, 100 individuals were tracked using Fitbit for a year (14–16). The study found significant variability in daily step counts, which correlated with EDSS scores. Notably, pwMS at all levels demonstrated wide variability. For example, those with an EDSS of 6 showed a broad range in the number of steps taken, from over 7,000 to merely 1,000, suggesting ambulatory variability under the same EDSS. The study, with an 82% retention rate, revealed that those with initial step counts below 4,700 had a fourfold higher risk of condition worsening. Even in participants with stable EDSS scores, notable step count changes were observed, indicating functional ability variations not detectable by standard clinical metrics. This highlights the potential of wearable technology in detailed MS disability monitoring and supports the inclusion of remote step count tracking as a novel outcome in MS research (15). In the same study (16), passive monitoring was employed to assess falls in pwMS, using questionnaires every three months. Falls are challenging to measure objectively, and literature suggests that self-reported recent falls are more predictive of future falls than complex measures (16). The study used average step counts and patient-reported assessments, specifically the MS Walking Scale-12 (MSWS-12), which evaluates how much patients believe MS affects their walking over four weeks. Findings revealed that those who reported a fall had lower average daily step counts and worse MSWS-12 scores compared to those who did not. It also identified MSWS-12 as a significant predictor of future falls within the year, demonstrating the efficacy of passive monitoring and patient-reported measures in understanding fall risks in MS (17, 18).

Urinary dysfunction and fatigue are significant yet complex challenges of MS management. Urinary dysfunction, impacting over 80% of pwMS, is frequently under-assessed and poorly represented by conventional measures. Fatigue, with its elusive nature and lack of an objective standard for measurement, primarily depends on PROs for evaluation. For instance, in a randomized placebo-controlled double-blind trial, the efficacy of three medications for fatigue was compared using the Modified Fatigue Impact Scale (MFIS) PRO as the primary endpoint (19, 20). The increasing use of smartphones enables frequent, real-time assessments of MS fatigue, potentially reducing recall bias associated with traditional, less frequent measures. Wearable devices may provide insights into fatigability and activity levels affected by subjective fatigue severity. Integrating remote fatigue measures with objective assessments of fatigability into a composite score could lead to a more comprehensive understanding of this symptom (21). Cognitive dysfunction, affecting up to 70% of people with MS, presents challenges in memory, attention, and executive functioning. Its subtle signs often lead to underdiagnoses. Cognitive assessments traditionally rely on PROs and tools like the SDMT (22–25). However, emerging digital technologies offer innovative, accessible ways for regular cognitive monitoring. Smartphone apps and online platforms enable real-time, user-friendly cognitive evaluations. Integrating these digital methods with conventional tests could improve cognitive impairment detection in MS, providing a fuller understanding of cognitive health changes over time (26, 27).

Looking ahead, the translation of this research into practical applications is envisioned, possibly through a closed-loop care model. This model is centered on the early detection of changes, prompt intervention, and sustained improvement in function. Continuous monitoring with multiple devices, although not the ultimate goal, is considered a valuable intermediary outcome, serving until a more comprehensive understanding and recognition of MS progression is achieved. However, moving forward some barriers have to be addressed. The digital monitoring needs to demonstrate appropriate early detection with reasonable sensitivity but very high specificity. False positives are an important issue especially for the patient with added burden of care and anxiety.

## 3 Lesson learnt from existing PROMs

### 3.1 PROMs global survey (a survey designed by and for pwMS)-WG1

The Working Group 1 of the Global PROMS Initiative focused on creating a survey designed by and for individuals with MS. The objectives were threefold: (1) to pinpoint and prioritize outcomes most significant to those affected by MS, (2) to offer guidance in identifying gaps within existing PROs, and (3) to establish a framework for developing new PROs crafted by individuals affected by MS. In 2020, the WG1 research team in close collaboration with pwMS members of the ECT, also comprising MS Societies’ representatives, shaped the survey’s content. The research WG1 team defined twenty-eight functional domains deemed most relevant to people with MS and conducted a pilot survey in 11 countries using six languages. Feedback received was incorporated to create the final survey, launched and disseminated worldwide.^5^ The survey’s objective aimed to empower individuals with MS to instigate positive change by assisting policymakers, researchers, and healthcare professionals in understanding what to monitor in MS and define outcomes in clinical trials. By the time of the ECF annual meeting (November 2023), over 2,300 surveys had been completed. At present the survey is closed and over 5,000 people living with the disease completed the online survey. Preliminary data analysis revealed a high interdependency among the 28 symptoms tested, with five clusters identified focusing on concentration, balance, anxiety, sensory changes, and sleep problems. The survey also highlighted gaps in current PROs, lacking the inclusion of symptoms considered important by people with MS, such as hearing impairment, weight control, social functioning, or immune vulnerability. The impact of side effects of therapies on people’s lives has also been raised as a significant and underestimated outcome measure that impacts on treatments’ decision. Additionally, the survey provided valuable insights into the personal experiences of pwMS for each symptom, as participants were invited to comment on the description of the 28 symptoms tested. A Delphi consensus study will utilize these qualitative analyses to modify the definition of each symptom, incorporating the patient’s voice into currently used clinical terminology.

### 3.2 Assessing the impact of multiple sclerosis symptoms – European MS platform

The European MS Platform (EMSP) is an umbrella organization for national MS organizations in Europe. EMSP’s strategic goals are to strive for better access to treatments, therapies and integrated care for pwMS across Europe, and to ensure that pwMS are involved in MS research and express their specific needs, that can then be integrated in the disease management.^6^ A previous recurrently conducted EMSP assessment, the MS Barometer, focused on the recording of the current management of MS across Europe. This survey demonstrated that there is low accessibility and reimbursement of Disease Modifying Treatments (DMTs) and symptomatic care and highlighted the need for a personalized approach for each person with MS.^7^

In order to understand which symptoms are affecting the daily life of pwMS, the EMSP launched the Impact of Multiple Sclerosis Symptoms (IMSS) survey. The IMSS survey aims to identify MS symptoms’ prevalence, clustering, burden and management, to investigate the relationship of the onset and severity of symptoms with the use of DMTs and symptomatic treatments, and to provide patient reported evidence that could enable the development of policies for QoL improvement.^8^ For this reason, data from 17,151 pwMS from 22 European countries were collected in 2023, via a digital platform. Collected data included, besides sociodemographic and disease-related parameters, the presence, frequency and severity of MS symptoms, the methods adopted by pwMS per symptom to care and manage their symptoms as well as the satisfaction and adequacy with how symptoms are being managed. The data are currently being analyzed. The IMSS survey aims to identify those symptoms that are not managed in a satisfying manner from the perspective of pwMS, which symptoms are the most debilitating for them, and which are affecting QoL the most. Thus, the results of this survey, together with the one developed by the PROMS initiative, is expected to provide recommendations for the use of relevant PROMs from clinical research to care.

### 3.3 The lack of scientific consensus on the use of PROs in MS clinical management-WG2

The integration of PROs in MS clinical care can enhance the understanding of individual experiences and contribute to personalized medicine. The lack of consensus on the use of PROMs in MS clinical management highlights the need for further research to identify PROMs used in clinical practice globally and reach a unified view for their targeted use for the different patients’ populations and individual. A literature review conducted in 2022 indicates that there is a limited number of PROs being collected and used in the clinical management of pwMS. Among 5,055 reviewed items from 2012 to 2021, only 37 met the selected criteria, emphasizing the limited attention to PROs in MS care. Notably, only 29% of the identified PROs were MS-specific, and conference abstracts accounted for a significant portion of the literature. Three measures of PRO encompassed a wide range of MS symptoms. Since more articles have been published in recent years, researchers have been attempting to determine whether the data might help recommend PROMs for different needs (1). The use in clinical practice of PROMs may exist but not make it to research publication or conference proceedings.

In MS care, PROs focus on patient-centric outcomes such as symptoms, disabilities, and QoL issues that patient experience. PROs in clinical trials and research, however, often prioritize clinician-centric data to meet statistical properties (validity, reliability etc.), primarily focusing on a single symptom. Work group 2 aims at emphasizing three primary roles for PROs in MS care: symptom detection and recording, treatment monitoring, and early prediction of disease progression.^9^ In this scenario, PROs enable a conversation in a Health Care Professionals (HCP)-pwMS interaction and promote shared decision making.

The variability of symptoms and QoL issues among people is typical of MS. Recognizing and treating the specific patterns experienced by individual patients is pivotal for personalized medicine. The variety of symptoms poses a significant challenge for creating PROs for monitoring disease progression. Personalized medicine refers to the identification and treatment of the specific symptoms and QoL difficulties that pwMS experience. SymptoMScreen (SMSS) is a PRO package that includes 12 functional domains most relevant to daily functions: mobility, dexterity, spasticity, body pain, sensation, bladder function, fatigue, vision, dizziness, cognition, depression and anxiety. SymtoMScreen uses 7-point Likert scales scoring for each functional domain. This impact questionnaire reflects functional changes that patients experience in daily life (28, 29). Examining data from SymptoMScreen reveals nuanced patterns in symptoms and progression, highlighting the significance of understanding individual variability (30).

The integration of PROs in MS care holds great promise for enhancing patient-centered care. However, their use in clinical trials and clinical practice has been quite limited because PROMs have been considered subjective and lacking of some fundamental qualities for a valid outcome measurements. The consequence has been the current lack of consensus and limited literature that necessitate continued research. The recommendations put forth by WG2 provide a roadmap for future initiatives, aiming at filling the gap between clinical research and care in the dynamic landscape of MS management. Additionally, aspects related to employment, including how work impacts on pwMS, absenteeism, presenteeism, and work quality, remain underexplored. Notably, the Work Productivity and Activity Impairment (WPAI) questionnaire represents a unique PRO that has seen some application and validation in MS contexts, suggesting an area ripe for further investigation (31–33). Identifying distinct groups of people with pwMS who share symptom patterns across functional domains and experiential knowledge, along with their interdependencies, will pave the way for a personalized application of PROMs from clinical trials to clinical practice and vice versa.

### 3.4 Screening of available registries and databases: preliminary analyses

The PROMS Initiative aims to conduct comprehensive screenings of available databases and registries to gather detailed insights into the content validity of existing PROMs. This effort will offer valuable information on how PROMs evolve along the disease trajectory and how they may differ across patient populations. The following available databases and registries case studies were presented at the 2023 Plenary Event meeting.

#### 3.4.1 UK MS registry

The United Kingdom Multiple Sclerosis Register (UKMSR) is a registry effort focused on multiple sclerosis that has been active since 2011 (34). It consists of an internet portal where pwMS have an active role in providing longitudinal data on disease and non-related information, including demographics of the users, characteristics of the disease at onset, symptoms, and PROs resulting from the regular periodic administration of online questionnaires. Clinical information is also collected from the NHS and linked to the online patient data. In this study, they used the permutation testing approach (35), this retrospective and prospective registry involved 15,976 pwMS and analyzed two physical disability PROs scales: the Multiple Sclerosis Impact Scale (MSIS-29 v2) motor component (36) and the Multiple Sclerosis Walking Scale (MSWS)-125 (37). Data were collected over 132 months, from May 2011 to April 2022.

The UKMSR study showed a significant effect of disease clinical subtypes on PROs and their interaction with disease duration. PROs worsened over time for all subtypes, and each subtype’s PROs were statistically distinct from others at all times (34). The study also found that PROs can indicate future transitions to progressive subtypes; individuals transitioning to progressive forms had higher score during the relapsing–remitting phase than those who did not transition highlighting early disability in potential progressive cases. Additionally, it is crucial to note that PRO results could predict further impairment or disability scored by traditional measures such as EDSS, underscoring the potential of PROs to provide early indicators of disease progression (38). Overall, PROs can effectively capture varying physical patterns, both improvements and worsening over time and across subtypes. Illustrating their utility as valid, cost-effective, and easily applicable tools for measuring MS’s physical impact.

#### 3.4.2 Swedish MS registry

Since 2000, the Swedish MS registry has provided a clinical interface that charts a patient’s medical history, including EDSS scores and treatments to monitor disease progression. The SMSreg Patient Portal, established in 2013 within the Swedish Neuro Registry, enables patients to oversee their health records, monitor their condition, and engage with healthcare providers for better self-management.

The Swedish MS registry (SMSreg) has collected extensive data from around 23,356 patients, including various metrics such as the EDSS, MSIS-29, SDMT, EuroQol-5D (EQ5D), Treatment Satisfaction Questionnaire (TSQ), Fatigue Scale for Motor and Cognitive functions (FSMC), and a comprehensive symptom checklist. This registry facilitates cross-validation of outcomes, longitudinal assessment for prediction, and links with national databases for comprehensive statistical analysis, including socioeconomics and comorbidities, enhancing MS research and treatment (39, 40).

The Swedish MS registry’s study confirms the correlation between the MSIS-29 and the EDSS, emphasizing the predictive value of early physical symptom changes for long-term MS management. Early initiation of disease-modifying treatments (DMTs) is linked to significantly better long-term physical outcomes in pwMS, demonstrated by EDSS and both the physical and psychological domains of MSIS-29 (41–44). The Swedish MS registry has demonstrated that PROMs can be successfully gathered by both patients and healthcare providers. The registry offers a rich and intricate array of data, presenting significant opportunities to cross-check and validate patient-reported outcomes. The importance and utility of current PROMs in the registry are highlighted as valuable tools for patient care and research.

#### 3.4.3 Italian Barcoding MS initiative

In 2000, the Italian collection of MS clinical data started at different Italian MS centers in the framework of the Italian Multiple Sclerosis Database Network (MSDN) (45). Within this frame, since 2013, the Italian MS Society representing pwMS (“Associazione Italiana Sclerosi Multipla”-AISM) together with its foundation (“Fondazione Italiana Sclerosi Multipla”-FISM) have been engaged in promoting and funding data sharing initiatives. In 2014, FISM, in collaboration with the Italian MS clinical centers, promoted and funded the creation of the Italian MS Register, with more than 80,000 patients currently included in the Register (46).

AISM and FISM supported several data-sharing initiatives in the last years, promoting the development not only of the Italian MS Register, but also of databases to study different aspects of MS. This included initiatives such as the Italian Network of NeuroImaging (INNI) (47), the PROgnostic GEnetic Factors in Multiple Sclerosis (PROGEMUS) (48), the Multiple Sclerosis and COVID-19 (MuSC-19) platform (49), and Patient-Reported Outcome Measures for MS databases (PROMOPRO-MS) (50). Integration of PROMs with other outcomes will help to enable the holistic approach needed to unmask progression early in the disease. Therefore, the ambition today is to go even further, with a data integration initiative called the ‘BARCODING MS’,^10^ This initiative has been launched in 2022, with the aim of developing an integrated database of clinical, genetic, imaging and PROMS, to create a figurative ‘barcode’ as a multidimensional picture of the person with MS. Each individual barcode represents a newly diagnosed person with MS in Italy. Using machine learning approaches, an algorithm will be developed to identify factors responsible for disease progression. This initiative will help researchers develop and recommend future personalized treatments for pwMS.

### 3.5 Toward value-based healthcare: the case of PROMOPROMS database-WG4

WG4 (Healthcare Systems and Policies) is working to generate guidelines for incorporating PROMs into healthcare systems. The main objective is therefore to improve patient satisfaction and elucidate reflections on the relationship between cost/efficacy of healthcare systems.^11^ This group is therefore characterized by a number of objectives such as translation of standardized data into a performance measure (PRO PM) that are adequate to capture the results most relevant for improving long term quality of life. Another important objective is to develop PRO PM in innovative reimbursement models as well as regulatory frameworks and clinical decision support algorithms. In this way, there could be a potential impact on tailoring healthcare for pwMS. Recent activities that are focused on this topic include development of semi-structural interviews at MS advocacy groups from high-income and middle-to-low-income countries in terms of the current uptake of PROs in their healthcare systems and their use as quality-of-care indicators for helping the policy decision-making. This included representatives (n = 6) of 5 MS organizations (Belgium, Denmark, UK, US and Canada) who were asked to participate based on their understanding of advocacy and their national policy landscape. Prior to carrying out a semi-structured interview, the official website of the subsequent MS organization was analyzed. Transcripts were analyzed and coded with NVivo.^12^ Currently, this preliminary analysis is complemented by additional interviews in underrepresented areas such as South Africa, Tunisia, Iraq, Egypt, Uruguay and Argentina. In this preliminary analysis, the authors have been able to demonstrate that MS organizations use both qualitative and quantitative data to advocate for change when negotiating with health policymakers, however, the knowledge and use of PROs by policymakers is still limited. Information gathered through patients’ organizations offers a unique viewpoint on perspectives of the families and patients that could later on influence policy. The unique methodology that is applied in such patients ‘organizations are focus groups, interviews, storytelling, surveys, and collections of personal experiences through websites and social media. Another important possibility offered by patients ‘groups is representativeness, although still some minorities are underrepresented even in this group of patients (51). However, further research and effort are required to identify the PROMs that can help policymakers understand the QoL of MS patients and caregivers, as well as the quality of care provided, in order to guide their decisions based on the identified needs and the concept of value-based healthcare.

## 4 The opportunity of digital PRO measures (e-health) – WG3

Digital tools have the potential to collect longitudinal data on several functional domains and help implement a more holistic approach to unmask unrecognized progression even early in the disease. Within the PROMS Initiative, the WG3 developed a “living,” updated library for eHealth tools, built on the MS Data Alliance Platform.^13^ The purpose of the catalog is to obtain an exhausting landscape analysis of eHealth tools available to pwMS, helping to identify gaps and inspire future directions for clinical validation and development. This dynamic, open resource is designed to support the PROMS Initiative to identify and select the most relevant eHealth tools able to measure over time functional domains that matter most to pwMS (WG1 survey). The focus of these tools is on the ongoing design, research, and utility for pwMS, with a particular emphasis on collecting PROMs. The eHealth catalog includes not only patient-reported outcomes – mainly in the form of questionnaires or numeric scales, but also patient-centered data collected actively or passively via smartphones or dedicated wearable sensors (52, 53). The latter allows to collect quantitative and qualitative data without requesting formal self-testing, such as measures of mobility during daily living activities. Moreover, a passive monitoring approach would allow reducing burden on the patient, providing more ecologic measures and improve adherence limits of procedures requiring active participation (54). Among studies investigating passive monitoring, Mobilise-D, a 5-year project (2019–2024) funded by the Innovative Medicines Initiative (IMI), aims at integrating digital mobility outcomes of gait to key clinical measures, advocating for regulatory and clinical endorsement (53). The project, mainly focused on objective measures of mobility that are relevant across several medical conditions (54), involves a substantial sample of 2,400 individuals across four key disease cohorts: Chronic Obstructive Pulmonary Disease (COPD), Proximal Femoral Fracture (PFF), Parkinson’s Disease (PD), and MS (53). For PwMS, the approach includes passive gait monitoring for one week using a wearable sensor, repeated at semi-annual intervals over two years. Mobilise-D is actively liaising with regulatory bodies, such as EMA and the FDA, to align the mobility assessment tools with clinical trial requirements and integrate them into mainstream clinical practice, reflecting a commitment to regulatory compliance and the advancement of healthcare technologies (53). Taking into account the difficulties in harmonizing measures obtained from different technologies (54), the clinical validation study included two different but methodologically equivalent wearable sensors, according to an agnostic device approach proposed in a technical validation phase (55). The use of sensors allowing to measure several gait parameters, beyond step count, may enhance the precision of mobility evaluations, offering insights critical for personalized healthcare strategies and better understanding of mobility-related disease progression or fall prediction. Within Mobilise-D, patient and public involvement and engagement (PPIE) activities have been developed to ensure that the technology proposed will be inclusive, clinically relevant and responding to the needs of the included groups (56).

In this era of digital transformation, through PROMS ECT, pwMS will be engaged to ensure the necessary level of acceptability required for successful selection and use of digital tools and related data collection. The catalog remains a dynamic resource, with the potential for expansion and refinement to better serve the eHealth community.

### 4.1 Smartphone-enabled mobile health: the promise and challenges in patient care

Mobile health (m-Health) technology holds considerable promise for enhancing patient care, offering a way to monitor health outside traditional clinical environments. Smartphones, equipped with an array of sensors, can conveniently gather data that more accurately reflect a patient’s daily life and abilities. This real-time health monitoring can potentially transform disease management and patient engagement in their own care. However, the integration of m-Health into routine practice poses its own set of challenges. Ensuring that patients accept these technologies and adhere to them, over prolonged periods, particularly when it comes to actively participating in tests and assessments, remains a substantial hurdle.

Adherence and retention rates are pivotal indicators of the effectiveness of m-Health applications, especially in unsupervised settings like remote research or consumer markets. These metrics tell of how consistently patients engage with health apps when not directly overseen by healthcare providers. While studies of different durations have demonstrated that sustained adherence to smartphone-based assessments can be achieved by a majority of participants in a supervised research context, reports from naturalistic settings have shown varying patterns of app usage over a 12-week period, overall indicating that user engagement can significantly drop off as time progresses. In a broader market context, retention rates reveal that within 90 days, healthcare apps retain only 31% of their users, and this figure decreases further over the course of a year (55, 56).

M-health technology, while poised to transform health monitoring and management, faces the persistent challenge of sustaining user engagement over time. Achieving long-term adherence to smartphone-based health assessments is multifaceted; although some platforms have shown success in short-term studies (57, 58), maintaining consistent participation over longer periods reveals divergent adherence patterns. Passive data collection methods via smartphones, leveraging the continuous recording and processing of accelerometer signals could ease participant burden and enhance adherence, while simultaneously yielding detailed, quantitative insights into patient motion behavior. Thus, developing strategies to bolster user’s acceptance and retention is essential to harness the full potential of m-health innovations.

### 4.2 Advancing patients acceptance of digital tools: the case of MSCopilot®

The MSCopilot®, a digital medical device explicitly developed for MS management, stands at the forefront of technological innovation in healthcare in the views of its developers. The device has undergone clinical validation involving 220 participants across 12 MS centers, demonstrating correlation with clinical standards MSFC and EDSS. This validation is backed by the publication of three peer-reviewed articles in reputable journals (59–61) see the new pubblication added in the reference part.

MSCopilot® has confirmed its clinical performance through post-market activities. In terms of research integration, MSCopilot® is part of three Phase II/III and long-term extension studies ensuring its ongoing evaluation and improvement. This involvement is complemented by a continuous scientific communication pipeline, disseminating findings through peer-reviewed journals and international conferences.

While MSCopilot® has demonstrated clear benefits within controlled clinical study environments, real-world application presents a different challenge, with a notable decline in user engagement over time. This discrepancy underscores the importance of persistent assessment and refinement of the tool to ensure it remains effective and relevant to patients’ daily needs, thereby sustaining their long-term engagement. In response to these challenges, a robust conceptual framework has been established to bolster the acceptance of digital health solutions. This strategic approach seeks to harmonize the device’s features with the actual health-related behaviors and routines of patients, thereby facilitating seamless incorporation into their daily management of MS and overall healthcare journey.

To foster its commitment to patient-centered care, the MSCopilot® is involved in several key studies aimed at enhancing its utility and validating its benefits. The “MSCopilot DETECT” is an international, multicenter, longitudinal study enrolling 314 patients to assess the device’s capacity to detect worsening disability. “MSCopilot LOTUS,” another significant initiative, is a large-scale longitudinal cohort study that includes 8,000 patients across the US and EU. It aims to gather real-world evidence on functional evolution and quality of life while also monitoring treatment satisfaction and modifications. Lastly, “MSCopilot BOOST,” a French multicenter study, involves 208 patients in a comparative, randomized, and longitudinal trial. It focuses on developing and validating a fatigue management module along with a physical activity recommendation engine to mitigate fatigability.

These case projects, presented during the 2023 Global PROMS Plenary Event, stimulated discussion on the effort needed to demonstrate and refine digital tools practical benefits, thereby fostering patient acceptance and enhancing the integration of digital tools into clinical care.

that the digital solutions provided align closely with individual patient needs and experiences.

## 5 Statistical challenges and perspectives

### 5.1 PRO measures and type 2 error in MS clinical trials

In the realm of clinical research, the statistical analysis encompasses a critical evaluation of potential errors, notably Type-2 errors, which represent instances where a true effect is falsely deemed non-significant. This type of error is particularly consequential in clinical trials as it can result in the erroneous rejection of a beneficial treatment due to the test’s failure to recognize a genuine effect. The implication that Type-2 errors are more significant than Type-1 errors underscores the importance of designing studies with sufficient power to detect true changes, ensuring that effective treatments are accurately identified and adopted into clinical practice. This error can stem from multiple sources, particularly the selection process, which includes the gathering and assessment of PROs. These PROMs are crucial as they capture patients’ experiential knowledge with the treatment. The development of these measures, as well as their performance, can significantly influence the occurrence of Type-2 errors. Furthermore, the analysis dives into context-specific issues that can skew results, such as the distribution properties of the data and the dependence of responses that may vary according to different patient groups or study conditions. The methodology employed in the analysis of PRO measure data is dissected, with an emphasis on selecting the right statistical methods and determining the appropriate level of data granularity to interpret results accurately.

To address these issues, a set of recommendations have been proposed, including the coordination of targeted programs dedicated to this specific area of work. Additionally, the routine implementation of advanced psychometric methods is advised to enhance measurement precision. Lastly, there is an opportunity to substantially improve the quality of PRO measurement, contributing to the overall improvement in clinical research methodologies.

### 5.2 New statistical approaches to analyze multiple outcomes

Clinical trials traditionally rely on a single primary endpoint and a single null hypothesis to determine the treatment effect, which is considered as an average effect across all enrolled participants. However, there has been a shift toward a more refined approach with the proposal of composite endpoints that allow for the consideration of multiple outcomes. This evolution in methodology underscores the need to move away from one-size-fits-all results toward a more personalized understanding of clinical efficacy that takes into account individual patient preferences and variations.

Innovative statistical methods are being introduced in clinical trials, particularly from the field of oncology, which allow for the evaluation of multiple outcomes through the creation of a prioritization list. This new approach requires patients to actively participate by determining their own personalized priority list for treatment outcomes and the PROMS survey will provide important insights in this direction. Consequently, the computation of the treatment effect is tailored, taking into account the individual patient’s prioritized outcomes, thereby introducing a level of personalization into the statistical analysis of clinical trials (62, 63).

This new method describes a treatment effect estimate called the “net chance of benefit,” which is calculated based on pairwise comparisons among all randomized patients within a clinical trial. Tailored for versatility, it assesses any variable type such as continuous data, time-to-event statistics, binary outcomes, and categorical variables and initially addresses single outcomes, yet its real strength lies in its adaptability to multiple outcomes, which are assessed according to their ranked significance. This approach hinges on a pairwise comparison of observed outcomes for each patient, assigning a score that reflects whether the treatment or control is favored, or if the result is neutral. The aggregate of these scores, normalized by the number of comparisons, yields a delta (Δ) value, which provides a quantifiable net benefit of the treatment. A Δ of zero suggests no discernible treatment effect, whereas a Δ of one indicates complete favorability toward the treatment over the control, and conversely, a Δ of negative one indicates the opposite.

This new method marks a step toward personalized healthcare by ensuring that treatment assessments in clinical trials consider the unique priorities of the different target patient populations, particularly through PROs, advocating for a more patient-reported and inclusive approach in research.

## 6 The industry’s perspective

From the industry perspective, utilizing PROs offers significant potential value and opportunities, especially in detecting subtle aspects of unrecognized disease progression that are not easily identified through traditional clinical measures. The industry is tasked with navigating the challenges associated with standardizing and integrating PRO measures, striving to facilitate and support a cohesive and unified approach that aligns with the broader healthcare objectives and regulatory standards.

PROs are positioned to significantly change the development of future MS drugs, spanning from research to clinical application. They are instrumental in validating new hypotheses about the course of MS and aiding in the development of biomarkers enabling the holistic approach needed to improve prognosis, prevent and treat progression and improve lives of pwMS. PROs also enable a more precise definition of unmet needs, thereby refining the identification of target patient populations. Additionally, they have the potential to evolve the conduct and efficiency of future clinical trials through more sensitive outcome measures and novel endpoints, optimizing trial design, duration, and size to better address disease progression. Lastly, PROs are key to capturing the wider effects of unrecognized disease progression on patient’s lives, including their role participation, work productivity, and health-related quality of life (HRQoL), which are critical factors for HTA bodies when evaluating the daily impact of the disease on patients.

PROs are crucial for the development of future MS drugs and collaborations involving multiple stakeholders and the active participation of all parties are essential for aligning the industry on the use and measurement of PROs.

## 7 The regulatory agency’s perspective (European Medicines Agency)

Patient Experience Data (PED) is collected through various patient engagement activities and methodologies, capturing the patients’ experience of their health status, symptoms, disease course, treatment preference, quality of life and impact of healthcare. PED can encompass quantitative data like PROs and PREMs and qualitative data collected via various patient engagement activities such as focus groups.

Enhancing patient relevance in evidence generation for medication approval and monitoring is a key priority of the EU Regulatory Network,^14^ which includes EMA. Despite progress, the systematic integration of PED into all aspects of medicine development and regulation is incomplete. There is a stakeholder agreement on PED’s importance for developing medicines and assessing their risks and benefits and calls for further guidance.

PED is crucial for neurology medicines development, reflecting patients’ unique insights into living with conditions and treatment impacts. During the evaluation phase, PED gathered using reliable and validated methods enriches knowledge to support primary and secondary endpoints in clinical trials, especially when harder and more quantifiable endpoints are not fully developed and have not reached maturity. For example, PED might yield insights for development of better measures of disease progression. In post-authorization phases, PED becomes a part of real-world data collection in registries, informing ongoing evidence generation for neurology products.

A multi-stakeholder workshop held in 2022 by EMA identified the need for a common understanding of PED in the EU, alignment among decision-makers, and cooperative action. It highlighted the necessity for clear regulatory guidance, transparency in PED assessment, and important links to the digitalization of health data to advance PED integration in medicines development and regulatory decision-making.

EMA is advancing on PED by building on the outcomes of the 2022 workshop. Collaboration with multidisciplinary experts across agencies and EU network is key to coordinating activities. EMA also supports the global development of PED and contributes to ongoing work on guidelines at the level of ICH.

In 2024 EMA will publish a reflection paper for public consultation, outlining an EU approach to generate, collect and analyze PED. All stakeholders will be able to contribute their views during the consultation process.

In parallel, EU regulators will explore how best to better reflect PED within scientific assessment reports including how PED is evaluated and used benefit/risk decision-making.

The digitalization of patient-generated health data is being integrated with ongoing activities within the European Health Data Space (EHDS) and is part of the Big Data work plan running from 2022 to 2025. The patient perspective is vital and will continue to be collected and utilized in these forums. Collaboration is underway with the EMA/HMA Big Data Steering Group (BDSG), and the BDSG work plan has been updated to include actions on PED.

## 8 Discussion and future strategic direction

The value of PRO and PROMs in identifying unrecognized progression and their potential as primary endpoints in clinical trials for treatment is acknowledged. There is a consensus on the importance of patient-generated data to complement traditional clinical measures and enhance the quantification of the patient’s experience. PROMs could redefine state-of-the-art in tracking or unmasking unrecognized progression through collaboration among clinicians, patients and other stakeholders. They may also revolutionize the development of future drugs by providing new hypotheses on MS course, defining unmet needs with greater precision, and improving efficiency in clinical trials. Despite their promise, the limitations in available PRO tools must be acknowledged.

To realize the added value of PROMs in identifying unrecognized progression, there is an essential need to progress research to deep phenotype pwMS experiential knowledge, dominant functional domains and their interdependencies, across disease journey and progression toward a personalized approach for the different target patients’ population. Measures should be differentiated based on each patient’s sociodemographic profile, inclusivity, and level of disease progression, while reflecting individual disease expectations. It is important to ensure that the aspects significant to patients align with the benefits they receive and that these are validated for their impact on enhancing quality of life. The meeting highlighted the need for further research toward a more targeted (personalized) use of PROMs for the different patients’ populations and individual from clinical trials to care. Deep understanding of pwMS experiential knowledge through WG1 survey and use of appropriate PROMs will contribute to personalized medicine. Targeted approaches with relevant PROMs will ensure clinical trials and clinical practice is aligned with patient values. Risk of confounding in clinical trials will be reduced through using PROMs that account for the underlying heterogeneity of disparate subgroups of pwMS. The current challenges in applying the same PROMs from clinical trials to care include the need for more incremental measurements beyond snapshots of function to capture subtle disease progression. Objective measures for monitoring functional domains over time could facilitate the passage of MS interventions through clinical trial phase II and III, improving access to care. Acceptance from all stakeholders, which include regulatory bodies like the FDA and EMA, pharmaceutical companies, healthcare providers, insurance agencies, and crucially, the patients themselves, is crucial. Health authority engagement is also vital for generating guidelines and recommendations with emphasis on the adoption of measures for clinical trials to be cascaded to clinical practice and vice versa.

To address the challenges of using PROMs from clinical trials in care, it is essential to overcome practical barriers such as the lack of valid, reliable, and responsive PRO measures with translations for global use. Enhancing engagement with health authorities can lead to better guidance and recommendations for adopting clinical practice measures within the MS community. Furthermore, clinical trials need to incorporate new methods that quantify treatment effects, taking into account multiple outcomes and patient preferences for a more patient-oriented approach, acknowledging that different patients have varying priorities.

To tackle the challenges in applying PROMs from clinical trials to care, collaborations are needed to validate PRO across various research initiatives, allowing data sharing without creating multiple instruments. Synergies with initiatives like the PROMS and EMSP surveys are crucial for comprehensive data collection. There is a need to address the risks of type-II errors in PROMs in clinical trials and ensure validity in fit-for-purpose PROMs, focusing on content validity. Advancing PROMs quality and incorporating routine advanced psychometric methods is essential for accurate outcome measurement.

The current digital era offers an abundance of tools, such as apps and wearables for data collection, allowing for active and passive tracking of changes over time. It is crucial to involve patients in the development of these digital devices, ensuring they report experiences and preferences that meet their needs for successful data collection and usage.

The PROMS initiative’s success hinges on developing a globally scoped, innovative survey in multiple languages for pwMS, aiming for widespread contributions. This data is instrumental for understanding functional domains and their interdependencies, guiding a Delphi study to pinpoint research gaps. Despite the absence of a scientific consensus on PROMs’ role, the initiative studies up to four measures that meet inclusive criteria for broad stakeholder acceptance.

The PROMS initiative has been mapping existing eHealth tools for monitoring various domains of interest. Work is ongoing to improve interoperability and patient acceptability of these tools. Additionally, an invitation to a November meeting in Madrid aims to boost the integration of PROMs in health policy both in Europe and globally. This meeting will advance the messages from the last working group of the PROMS initiative focused on healthcare systems.

Despite differences in point of view, during the 2023 Global PROMS Plenary Event, relevant stakeholders agree that PROMs, and the PROs that they yield, have not reached their full potential for delivering benefits to pwMS and the healthcare continuum. Reaching a consensus, rather than a standardization (the meeting indicated that One Size Does Not Fit All) of the PROMs’ use necessitates the establishment of appropriate incentives and enhanced multi-stakeholder collaboration. The PROMS Initiative represents indeed the pre-competitive platform promoted with the aim to reach a consensus, a unified view on PROMs, which also helps to prevent the proliferation of scales and facilitate the sharing of data. Addressing practical barriers to the use of PRO measures is also crucial; first enabling research with pwMS input is instrumental to ensure the availability of PRO measures that are reliable and with high content of validity (also including versions in multiple languages) (51). This of course also raises the point that the other stakeholders (e.g., regulators and payers) must be involved early in discussions on how to generate PED that can be acceptable for different regulatory and reimbursement purposes, tailored to the different target populations and welcome the opportunities provided by the digital transformation of research and healthcare (e.g., collection of real-world evidence through digital devices). Overall one of the primary objective of the PROMS Initiative is to qualify scientifically the active and passive PROMs that will be identified with an accurate validation process that meets the expectations of all the relevant stakeholders. This is in line with the participatory governance of the PROMS Initiative. As far as digital tools, we acknowledge the potential difficulty of implementing them globally and the accessibility problems will be a key criteria for the selection of the specific algorithm.

## 9 Closing note from PwMS: designing with the end in mind

The field of PROs and PROMs is developing at speed and PROs have the potential to facilitate communication with a person’s clinical team and increase involvement in decision-making for treatment and care. At the same time, there are several notes of caution. There are known differences in which PROs/PROMs are used (and how they are used) between clinical trials, clinical practice, and healthcare governance systems. This heterogeneity introduces important ethical considerations that are influenced by PRO content and implementation context. One must ensure ethical preparedness to emerging innovations in PRO usage that align with moral values and support the welfare of pwMS. Technological advances in monitoring and data capture, and the likely increasing influence of artificial intelligence systems, means there is a need for horizon scanning to embed ethical principles around PROs. We must fully consider how PROs could have a negative impact and drive further health inequalities. If there is a potential to empower, we must consider the potential to disempower. If there are opportunities to increase system efficiencies, we must explore the potential for increased burden for pwMS. If PROs can enable increases in day-to-day support in living with the condition, how can we explore the potential for support to be taken away? Moreover, enhanced global connectivity means data can be shared easily between stakeholders, so it is imperative that data sharing is appropriate, proportionate, and subject to informed consent. Co-designing MS-specific PROs with pwMS is encouraged to ensure that healthcare systems look more broadly than pharmacological or behavioral interventions to reduce or monitor functional impairments. PROs should also be used to inform how a pwMS participates in daily life. PROs should demonstrate an appreciation of the wider determinants of health and pwMS’ opportunities to explore meaningful contributions within the communities they live.

### Member of the PROMS Initiative Scientific Committee and Working Groups

**The PROMS Initiative Scientific Committee**: Patrick Vermersch (co-chair); Paola Zaratin (co-chair); Maria Pia Amato; Giampaolo Brichetto; Paolo Cortesi; Gary Cutter; Gilles Edan; Emma Gray; Anne Helme; Jeremy Hobart; Robert Hyde; Usman Khan; Letizia Leocani; Lorenzo Mantovani; Robert McBurney; Iris-Katharina Penner; Valentina Tageo (program manager). **PROMS Initiative Working Group – Research, Validation and Development**: Maria Pia Amato (co-leader); Giampaolo Brichetto (co-leader); Alexey Boyko; Nancy Chiaravalloti; Brigit de Jong; Sonya Eremenco; Peter Feys; Luca Ghirotto; Giusy Iorio; Terrie Livingston; Julia Morahan; Roshan Nair; Christel Naujoks; Ainhoa Ruiz Del Agua; Krzysztof Selmaj; Alessandra Solari; Ingrid Van der Mei; Susanna van Tonder; Bassem Yamout. **PROMS Initiative Working Group – Clinical Management**: Gilles Edan (co-leader); Jeremy Hobart (co-leader); Robert McBurney (co-leader); Gary Cutter (co-leader); Iris-Katharina Penner (co-leader); David Cella; Jana Hlavácová; Robert Hyde; Christine Lebrun; Paul Kamudoni; Moksharif Nasrulloeva; Jaume Sastre Garriga; Aksel Siva; Helga Weiland; Angela White. **PROMS Initiative Working Group – e-Health**: Letizia Leocani (co-leader); Robert Hyde (co-leader); Valerie Block; Johan van Beek; Ivan Bozin; Luca Capezzuto; Richard Dobson; Joanna Dronka-Skrzypczak; Marcus Dsouza; Marni Hall; Lotte Geys; Ludwig Kappos; Daphne Kos; Johannes Lohrscheider; Paul Matthew; Liesbet Peeters. **PROMS Initiative Working Group – Health Care System**: Usman Khan (co-leader); Paolo Cortesi (on behalf of Lorenzo Giovanni Mantovani co-leader); Edgardo Cristiano; Benjamin Davis; Pietro Ferrara; Nupur Greene; Bruno Musch; Guy Peryer; Alexandra Piraino; Per Soelberg Sørensen; Bart Van Wijmeersch; Lisa Ye.

## Author contributions

PZ: Conceptualization, Writing – review & editing. SS: Writing – review & editing. MeS: Writing – review & editing. VR: Writing – review & editing. JS: Writing – review & editing. AT: Writing – review & editing. GB: Writing – review & editing. TC: Writing – review & editing. AH: Writing – review & editing. UK: Writing – review & editing. RMc: Writing – review & editing. GP: Writing – review & editing. HW: Writing – review & editing. PB: Writing – review & editing. MB: Writing – review & editing. VB: Writing – review & editing. LuC: Writing – review & editing. LoC: Writing – review & editing. PC: Writing – review & editing. GaC: Writing – review & editing. LL: Writing – review & editing. H-PH: Writing – review & editing. JaH: Writing – review & editing. JeH: Writing – review & editing. KI: Writing – review & editing. PK: Writing – review & editing. RMi: Writing – review & editing. PM: Writing – review & editing. XM: Writing – review & editing. LP: Writing – review & editing. MaS: Writing – review & editing. ST: Writing – review & editing. AW: Writing – review & editing. GiC: Conceptualization, Writing - review & editing. PV: Conceptualization, Writing - review & editing.

## Footnotes

^1^ https://proms-initiative.org/

^2^EU4 Health program program 2021–2027 – a vision for a healthier European Union. Available from: https://health.ec.europa.eu/funding/eu4health-programme-2021-2027-vision-healthier-european-union_en.

^3^Food and Drug Administration (FDA) Guidance for industry patient-reported outcome measures: use in medical product development to support labeling claims. December 2009. Available at: https://www.fda.gov/media/77832/download.

^4^European Medicines Agency (EMA) Reflection paper on the use of patient reported outcome (PRO) measure in oncology studies. EMA/CHMP/292464, June 2014. Available at: https://www.ema.europa.eu/en/documents/other/appendix-2-guideline-evaluation-anticancer-medicinal-products-man_en.pdf.

^5^Measuring what matters to people with MS. https://proms-initiative.org/measuring-what-matters-to-people-with-ms/.

^6^European Multiple Sclerosis Platform (EMSP): https://emsp.org.

^7^MS Barometer 2020, European Multiple Sclerosis Platform: https://msbarometer.eu/2020.

^8^Impact of Multiple Sclerosis Symptoms (IMSS), European Multiple Sclerosis Platform: https://emsp.org/projects/impact-of-multiple-sclerosis-symptoms-imss/.

^9^ https://www.prognosisresearch.com/progress-framework

^10^ https://emsp.org/news/barcoding-ms/

^11^WG4 Healthcare System and Policies. Available at: https://proms-initiative.org/wg4/.

^12^ https://lumivero.com/products/nvivo/

^13^ https://msda.emif-catalogue.eu/

^14^European medicines agencies network strategy to 2025.
https://www.ema.europa.eu/en/documents/report/european-union-medicines-agencies-network-strategy-2025-protecting-public-health-time-rapid-change_en.pdf

## Glossary

ADL: Activities of daily living
AISM: Italian MS Society
CVS: Clinical validation study
COPD: Chronic obstructive pulmonary disease
DMOs: Digital mobility outcomes
DMTs: Disease-modifying treatments
ECT: Engagement coordination team
ECTRIMS: European Committee for Treatment and Research in MS
EDSS: Expanded Disability Status Scale
EHDS: European Health Data Space
EMA: European Medicines Agency
EMSP: European MS Platform
EQ5D: EuroQoL-5D
FDA: Food and Drug Administration
FISM: Italian MS Foundation
FSMC: Fatigue Scale for Motor and Cognitive Functions
HRQoL: Health-related quality of life
HTAs: Health technologies assessments agencies
IMI: Innovative medicines initiative
IMSS: Impact of MS symptoms
INNI: Italian Network of NeuroImaging
MFIS: Modified Fatigue Impact Scale
MS: Multiple sclerosis
MSDN: MS database network
MSFC: MS functional composite
MSIF: MS international federation
MSIS-29: MS Impact Scale-29
MSW-12: MS Walking Scale-12
MSW-125: MS Walking Scale-125
MuSC-19: MS and COVID-19
NEDA: No Evidence of Disease Activity
OMOP: Observational Medical Outcomes Partnership
PD: Parkinson’s Disease
PED: Patient Experience Data
PFF: Proximal Femoral Fracture
PIRA: Progression Independent of Relapse Activity
PREMs: Patient Reported Experience Measures
PROs: Patient Reported Outcomes
PROGEMUSPROMS: Prognostic Genetic Factors in MS
PROMS: Patient Reported Outcome for MS Initiative
PROMs: Patient Reported Outcome Measures
PROMOPRO-MS: Patient-Reported Outcome Measures for MS databases
pwMS: Individual living with MS
QoL: Quality of life
SDMT: Symbol Digit Modawlities Test
SMSreg: Swedish MS registry
SMSS: SymptoMScreen
SPI2: Randomized, double-blind, placebo-controlled, phase 3 trial for high-dose biotin (MD1003) in patients with progressive MS
TSQ: Treatment Satisfaction Questionnaire
UKMSR: United Kingdom MS Register
